# Implementing the ICOPE program amongst community-dwelling older adults in Singapore: a multistage implementation study protocol

**DOI:** 10.3389/fpubh.2025.1672852

**Published:** 2025-12-12

**Authors:** Quan Wang, Li Feng Tan, Weng Mooi Tan, Reshma Aziz Merchant, Nick Sevdalis

**Affiliations:** 1 Centre for Behavioural and Implementation Science Interventions (BISI), Yong Loo Lin School of Medicine, Clinical Research Centre, National University of Singapore, Singapore, Singapore; 2 Healthy Ageing Program, Alexandra Hospital, Singapore, Singapore; 3 Division of Geriatric Medicine, Department of Medicine, National University Hospital, Singapore, Singapore; 4 Department of Medicine, Yong Loo Lin School of Medicine, National University of Singapore, Singapore, Singapore; 5 Integrated Health Division, MOH Office for Healthcare Transformation, Singapore, Singapore

**Keywords:** integrated care, older people, intrinsic capacity, implementation evaluation, mixed-methods study

## Abstract

**Background:**

The global population of older adults has significantly increased in recent years, presenting substantial public health challenges. This demographic shift underscores the necessity for effective interventions such as the Integrated Care for Older People (ICOPE) framework, which aims to preserve functional ability and intrinsic capacity. While ICOPE has been implemented and evaluated primarily by clinical practitioners, demonstrating its efficacy in detecting declines and initiating care, the application within community multidisciplinary teams remains underexplored. This study aims to prospectively examine the implementation of the ICOPE program and identify key factors influencing it within a community setting, through the involvement of multiple stakeholders.

**Methods:**

This longitudinal mixed-methods study will evaluate ICOPE implementation among community-dwelling older adults in Singapore, assessing reach, efficacy, adoption, implementation, and maintenance. Data will be collected at baseline and 3-, 6-, and 12-month follow-ups using surveys, electronic health records, interviews, and focus groups. Descriptive statistics will analyze trends, while qualitative data will explore implementation factors.

**Discussion:**

This multistage evaluation of ICOPE implementation will identify barriers and facilitators affecting its adoption and sustainability among healthcare providers and community-dwelling older adults. The findings may inform strategies for scaling ICOPE in resource-limited settings and support policy development to promote effective aging-in-place programs.

## Background

The global population of older adults has significantly increased in recent years, presenting substantial public health challenges. As of 2020, the number of individuals aged 60 and older reached 1 billion, and projections indicate this figure will increase to 1.4 billion by 2030 due to rising life expectancy and declining fertility rates (1, 2). This demographic transition underscores the critical importance of preserving functional ability and intrinsic capacity (IC)—a composite measure of physical and mental capabilities—to enhance health-related quality of life, minimize dependency, and mitigate excessive utilization of medical resources among older adults (3–5).

Recognizing these trends, the World Health Organization (WHO) introduced the Integrated Care for Older People (ICOPE) framework in 2017 to promote a person-centered, integrated approach that emphasizes early detection and proactive management of declining capacities (6). Updated in ICOPE 2.0, the four-step process—basic assessment, in-depth assessment, personalized care plan, and implement and monitor—now includes lifestyle advice and community-based health care initiatives in Step 1 to address IC losses (7). By embedding lifestyle interventions and engaging community stakeholders from the outset, ICOPE 2.0 strengthens continuity of care, empowers older adults to participate actively in maintaining IC, and bridges the divide between health and social services. However, despite growing evidence of its effectiveness in identifying IC impairments, the implementation and evaluation of the full ICOPE pathway within real-world community settings remain limited (8).

The latest ICOPE version emphasizes engaging a diverse array of community stakeholders at each step to improve access to social and healthcare services, deliver direct community-based support, and empower older adults to actively participate in decisions about their health (7). Implementing ICOPE in community settings poses significant challenges, particularly in fostering multidisciplinary cooperation and aligning community stakeholders (9). Effective delivery necessitates collaboration among healthcare professionals, social workers, and community stakeholders. However, the lack of standardized communication protocols often results in fragmented care and inconsistent implementation of integrated care (10). Additionally, differing professional priorities—such as a clinical emphasis on health outcomes versus a psychosocial focus on well-being—coupled with limited interdisciplinary training, further undermine cohesive teamwork (8, 10, 11). These obstacles can impede the integration of essential community resources, like infrastructure and personnel, thereby limiting the overall impact of the framework (7, 8).

Adherence to ICOPE interventions among older adults poses additional barriers to effective community implementation. These can be grouped into individual/community-level, affecting people with frailty and their immediate networks and environments; professional-level, affecting the people providing care, either in a professional or unpaid/volunteer capacity; and lastly system-level barriers, which reflect wider contextual factors. More specifically, individual factors like low health literacy and limited awareness reduce older adults’ engagement in screening and follow-up activities, while physical and cognitive impairments, such as mobility limitations or memory decline, restrict participation in recommended interventions (12, 13). At the level of care provision, hesitation to disclose personal issues to community care providers who may be perceived as “strangers,” coupled with cultural beliefs and practices, and lack of role clarity among professional and unpaid providers of care may shape help-seeking behaviors and potentially delay in diagnosis and treatment among specific ethnic groups (14). Lastly, in modern multi-ethnic countries like Singapore, additional systemic challenges arise. Language barriers can impede older adults from establishing contact and rapport, especially when exacerbated by self-ageist attitudes that diminish their sense of empowerment during digital programs (15).

Taken together, these challenges emphasize the need for a multistage implementation evaluation to systematically identify barriers and facilitators among older adults, and also amongst the professional groups involved in planning, implementing and following up on ICOPE results within Singaporean community settings. Such evidence can enhance the fidelity of ICOPE framework’s delivery (by providers) and receipt (by older adults), thereby examining its efficacy in ethnically diverse community contexts and supporting its scalability. This study aims to evaluate the implementation outcomes of the ICOPE program across its stages and identify barriers and facilitators in a multi-ethnic, Asian community setting. The specific objectives are: (1) to assess ICOPE framework implementation by evaluating reach, efficacy, adoption, implementation fidelity (in delivery and receipt), strategies, and maintenance across phases, tracking trends over time; (2) to investigate factors influencing ICOPE framework delivery and uptake as barriers or facilitators; and (3) to develop evidence-based strategies to enhance ICOPE framework’s scalability in low-resource community settings.

## Methods

### Theoretical approach

The present study integrates four theoretical frameworks to assess implementation outcomes and factors affecting implementation comprehensively. These are outlined in the sections that follow.

### Reach, effectiveness, adoption, implementation, maintenance (RE-AIM)

RE-AIM serves as the primary framework for evaluating ICOPE framework implementation outcomes across all study phases, encompassing reach (resident participation), efficacy (health impact on residents), adoption (uptake by providers and residents), implementation (fidelity in delivery and receipt, along with strategies), and maintenance (sustainability and penetration among providers) (16).

### Diffusion of innovations (DoI)

DoI guides the evaluation of ICOPE’s initial adoption and influencing factors at T0 (baseline) and T2 (6 months) by analyzing its spread through adoption stages (knowledge, persuasion, decision, implementation, confirmation) and social systems, focusing on innovation attributes (e.g., advantage, complexity) and adopter types (e.g., early adopters, laggards) for providers and residents (17).

### Normalization process theory (NPT)

NPT examines needs and suggestions for sustaining ICOPE at T3 (12 months) by evaluating its integration into routine practice and daily care through four constructs, coherence (sense-making), cognitive participation (engagement), collective action (operational work), and reflexive monitoring (appraisal), for providers and residents (18).

### Consolidated framework for implementation research (CFIR)

CFIR informs the interpretation of ICOPE barriers and facilitators through five domains, including intervention characteristics (ICOPE features), outer setting (community context), inner setting (team dynamics), individual characteristics (provider and resident traits), and process (implementation steps), highlighting challenges and enablers (19).

By integrating these frameworks, this study grounds its findings in established implementation science theories, offering a comprehensive understanding of ICOPE framework’s initial adoption and long-term sustainability in community settings. RE-AIM serves as the primary framework to evaluate ICOPE framework implementation across all stages, assessing reach, efficacy, adoption, implementation fidelity, strategies, maintenance, and penetration. DoI examines early adoption by analyzing how providers and residents perceive and adopt ICOPE framework during the initial implementation stages. NPT explains how ICOPE integrates into routine practice and daily care among providers and residents in later stages. CFIR identifies contextual factors shaping these outcomes, including intervention characteristics, organizational dynamics, and community conditions.

### Study design and setting

**Figure 1:**
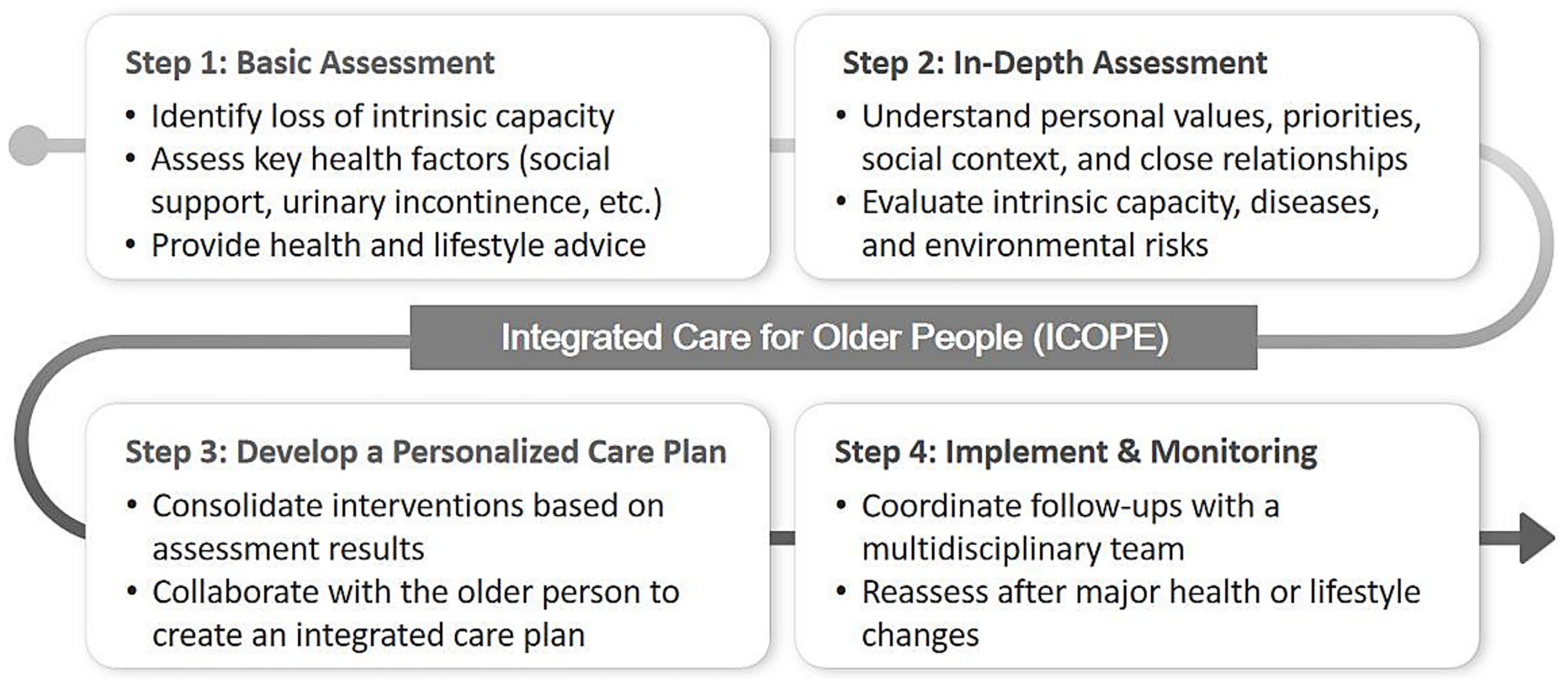
Steps of the ICOPE pathway (7).

This longitudinal mixed-methods study evaluates the implementation of the WHO ICOPE framework over 12 months in Boon Lay, a residential precinct in Singapore’s Western Region. Boon Lay was purposefully selected as one of the pilot sites for the Movements for Health Program, under Singapore’s broader Health District and Age Well SG initiatives, which integrate healthcare, social, and community services to promote healthy ageing in place. The precinct’s well-established network of Active Ageing Centres (AACs), community health posts, and regional health-system partnerships provides an enabling environment to operationalize the full ICOPE pathway across clinical and community interfaces. In addition, Boon Lay’s multi-ethnic demographic profile closely mirrors Singapore’s national composition, enhancing the study’s representativeness and translational relevance. Approximately 22.6% of its residents are aged 60 and above, with a demographic distribution of Chinese (64.9%), Malay (23.3%), and Indian (9.2%) (20). This closely aligns with Singapore’s overall population distribution, where 25.1% are above 60 years old, and ethnic proportions are Chinese (74.3%), Malay (13.5%), and Indian (9.0%) (21). As illustrated in Figure 1, the study covers all four ICOPE steps and is conducted across a network of community and healthcare settings that embody Singapore’s integrated primary care and community-oriented model, supported by the ICOPE Monitor app (7):

- Step 1 Basic Assessment: Conducted in Boon Lay community spaces (e.g., community clubs) and Active Ageing Centres (AACs), leveraging Singapore’s existing community infrastructure to facilitate initial screening.
- Step 2 In-Depth Assessment: Takes place at National University Polyclinics (NUPs), Community Health Posts (CHPs), and hospitals within the National University Health System (NUHS)—Ng Teng Fong General Hospital, National University Hospital, and Alexandra Hospital—highlighting Singapore’s tiered healthcare system.
- Step 3 Develop a Personalized Care Plan: Delivered at NUPs, CHPs, AACs, and NUHS facilities, with additional community support from the Health Promotion Board (HPB), showcasing multidisciplinary collaboration and community integration.
- Step 4 Implement & Monitoring: Combines remote monitoring via the ICOPE Monitor app with in-person visits in Boon Lay, coordinated by NUHS study team, reflecting Singapore’s emphasis on digital health solutions and comprehensive follow-up.

### Participants

Healthcare providers involved in the ICOPE care pathway in Boon Lay will be recruited using purposive and snowball sampling, targeting participants from eight professional groups: primary care providers, specialists, allied health professionals, social workers and community support staff, community engagement workers, health promotion and public health personnel, pharmacists, and those in coordination and policy support roles. Table 1 provides a detailed breakdown of the functions, roles, and corresponding professionals for each ICOPE step, illustrating their contributions to screening, assessment, care planning, and follow-up within Boon Lay’s community and healthcare settings.

**Table 1:** Roles and functions of healthcare providers in the ICOPE care pathway Singapore context.

Steps	Functions	Roles suggested by WHO	Singapore corresponding professionals
Step 1: Basic assessment and community level interventions	• Conduct basic assessments for loss of IC • Provide lifestyle advice and screening for CVD risk factors	Trained community health workers, community volunteers, and social workers or other health workers	Trained AAC staff, community health workers, micro-jobbers, volunteers, CHP Staff (e.g., nurses)
	• Organize events and encourage participation. • Raise awareness about healthy aging • Support community-based healthcare for IC loss	Community health workers and other trained community stakeholders	AAC/CHP/HPB/MOHT staff, SGA, volunteers, CCA, PHW
Steps 2: In-depth assessment	• Investigate and confirm IC impairments • Evaluate underlying diseases and risk factors • Assess social and physical environments	Health workers at primary care facilities	Geriatricians, dietitians, ophthalmologists, psychogeriatricians, NUP/CHP/polyclinic staff, nurses, pharmacists, PT, OT, ST
	• Ensure access to assessments via transport and follow-up support	Community stakeholders	Community organizations, volunteers, RSW
Step 3: Developing a personalized care plan	• Review findings and discuss opportunities to improve IC, functional ability, health, and well-being • Agree on interventions and finalize/share care plans.	Multidisciplinary team in primary care	CHP, Trained AAC staff, geriatricians, dietitians, psychogeriatricians
	• Provide social care and support • Map local resources to inform care plans	Community social care systems and social workers	NGO partners, AAC/ MOHT, RSW, volunteers, community organizations, NUHS Regional Health System Office
Step 4: Implementing and monitoring	• Coordinate and collaborate on care delivery • Follow up via in-person meetings, phone calls, or video calls	Multidisciplinary medical team	CHP staff, NUP
	• Manage social care services (e.g., day centers, meal delivery) • Promote social engagement (e.g., group activities) • Facilitate healthcare access (e.g., transport, outreach)	Community social care systems and social workers	NGO partners, volunteers, AAC/HPB/RSW, SGA, CCA, community organizations

AAC staff, active ageing centre staff (e.g., care managers, NTUC Health); CCA, community care associates; CHP staff, community health post staff (e.g., nurses); Dietitians, dietitians/nutritionists; GP, general practitioners; HPB staff, health promotion board staff; MHTO staff, multidisciplinary health project team staff (e.g., from Ministry of Health Transformation Office); IC, intrinsic capacity; NGO partners, non-governmental organization partners [e.g., Community Mental Health Intervention Team (COMIT), Community Resource, Engagement and Support Team (CREST). These are teams that provide support and care for the people at risk of or with cognitive or mental health issues]; NUP staff, network of upstream providers staff (e.g., primary care doctors); OT, occupational therapists; PHW, public health workers (e.g., health educators); PT, physiotherapists; RSW, registered social workers; SGA, silver generation ambassadors; ST, speech therapists.

Inclusion criteria for healthcare providers include: (1) at least one year of continuous professional or volunteer experience directly serving adults aged 60 and older in a healthcare, community, or technical support role; (2) willingness to complete surveys and interviews throughout the 12-month study; and (3) proficiency in English or Mandarin, confirmed by self-report and a brief screening conversation. Exclusion criteria include: (1) enrollment in another conflicting research study; or (2) unavailability to complete all required surveys or interviews.

Older residents will be recruited using simplified stratified sampling to ensure diversity in ethnicity (Chinese, Malay, Indian), age (60 ~ 74, 75+), and gender (male, female). Inclusion criteria include: (1) being aged 60 or older; (2) residing in Boon Lay for at least 12 months during the study; (3) demonstrating proficiency in English or Mandarin, confirmed by self-report and a brief screening conversation. Exclusion criteria include: (1) inability to provide informed consent due to cognitive or legal impediments; (2) current participation in another conflicting research study; or (3) unavailability to complete all required study assessments over the 12-month period.

### Sample size

For the quantitative component, sample sizes were calculated for different implementation components based on the RE-AIM framework. (1) For reach, the sample size was calculated using the cross-sectional design formula ( $n=\frac{Z^{2}\times P\times (1-P)}{E^{2}}$ ), based on a 67.8% prevalence of IC impairment among older community-dwelling adults (22), a 95% confidence level, and a 5% margin of error, rounded up from 336 to ensure adequate representation. (2) For efficacy, a paired t-test will be used with the effect size based on a standardized mean difference (SMD) of 0.36, derived from a meta-analysis by Liu et al. (23), and assuming a two-sided test with a significance level of 0.05 and a power of 0.80, the required sample size is 152 participants, randomly selected from those who complete the 12-month follow-up. (3) For adoption, fidelity, and maintenance, descriptive statistical analysis will examine implementation trends across four time points. A target sample of 10 providers will be recruited, starting with 8 participants from diverse professions to account for a 25% attrition rate, ensuring sufficient data. For older residents, at least 80 participants will be recruited through stratified random sampling across 12 strata (3 ethnicities × 2 age groups × 2 genders), ensuring at least 5 participants per stratum, with an initial sample of 60 to adjust for a 25% attrition rate.

For qualitative data, 12 ~ 16 providers and 15 ~ 18 residents will be interviewed at T0 and T2, with sampling continuing until thematic saturation is reached, ensuring diverse perspectives across ethnicity, age, and gender (24). At T3, at least two focus groups per group (4 ~ 8 participants each, totaling 8 ~ 16 participants) will be conducted (25). This mixed-methods sampling strategy aims to optimize descriptive breadth and qualitative depth while staying within resource constraints.

### Data collection process

The data collection process will be conducted at two primary levels, healthcare providers and older residents, across four distinct time points, baseline (T0), 3-month (T1), 6-month (T2), and 12-month (T3). Data collection integrates quantitative methods [i.e., surveys, observations, and electronic health record (EHR) reviews] with qualitative approaches (semi-structured interviews and focus groups) to capture a comprehensive view of implementation determinants and their outcomes over time (see Table 2).

**Table 2:** Study design across implementation phases.

Phase	Time point	Focus	Data collection methods
T0 (Pre-implementation)	Baseline	Initial perceptions	Interviews, surveys
T1 (Early Implementation)	3 Months	Early adoption and challenges	Surveys, observations, EHR data
T2 (Mid-Implementation)	6 Months	Fidelity, influencing factors	Surveys, interviews, EHR data, observations
T3 (Post-Implementation)	12 Months	Sustainability, penetration	Surveys, focus groups, EHR data, observations

EHR, electronic health record.

Quantitative data will be collected using standardized tools at specific intervals: surveys from providers and residents at T0 and T2 to evaluate initial perceptions and adoption of the ICOPE program among early adopters; followed by additional surveys, electronic health record data, and structured observations from T1 to T3 to assess implementation fidelity in both delivery and receipt; as well as implementation strategies, with sustainability and program penetration specifically evaluated at T3.

Qualitative data collection will progress across phases, starting with DoI-based interviews at T0 and T2 to examine adoption factors, followed by NPT-guided focus groups at T3 to assess sustainability needs. Bilingual researchers will conduct all assessments in English, Malay or Mandarin Chinese, based on participant preference, ensuring accessibility. Quality assurance in data collection will be ensured through researcher training in mixed methods data collection and analysis, as well as the specific frameworks applied within the study, prior to joining the study (e.g., through formal taught courses, or short professional development courses); and through study-specific calibration of the use of the data collection instruments, including interview topic guides and scales. The scales and interviews will be piloted with a small number of participants (e.g., 2 residents and 2 providers) to ensure feasibility, and the data and process of data collection will be reviewed jointly with a senior scientist (NS), to ensure inter-researcher consistency and resolve any identified problems. Data collection will then be carried out by individual researchers until study completion. Interviews and focus groups will be digitally recorded. All digital recordings will be deidentified and transcribed verbatim, in English, for subsequent analysis. Any data collected in the Chinese language will be translated/transcribed into English by the research team for the purposes of analysis. Recordings and transcripts will be digitally stored on a secure NUS server, with access to the data by designated researchers, as per the study’s ethical approval requirements.

This staged, mixed-methods approach allows for longitudinal tracking of implementation trends and triangulation of findings, providing a comprehensive evaluation of ICOPE’s community-based implementation.

### Measures

#### Implementation measures

Implementation measures in this study are evaluated using the RE-AIM framework, encompassing reach, efficacy, adoption, implementation, and maintenance (26). Data collection procedures are outlined in Table 3, with questionnaires and tools detailed in Supplementary file 1.

**Table 3:** Implementation measures across study timepoints.

RE-AIM structure	Measures	Tools for providers	Tools for residents	Timepoints
Reach	Participation rate of ICOPE program among residents	N/A	EHR data (enrollment data)	T0
Efficacy	Effectiveness of managing IC in residents	N/A	EHR data (ICOPE tool)	T0, T3
Adoption	Intention to use ICOPE among providers and residents	Adapted 8-item TFA for providers	Adapted 8-item TFA for residents	T0, T2
Implementation	Fidelity in delivery and receipt, and strategies of adaption	Direct observation, TIDieR checklist, and ERIC checklist	EHR data (self-monitor through ICOPE app, dietary platform data), IPAQ-SF	T1-T3
Maintenance	Extent to which the intervention is sustained and institutionalized in long-term	23-item NoMAD and 12-item SCIROCCO	N/A	T3

RE-AIM, reach, effectiveness, adoption, implementation, maintenance; IC, Intrinsic Capacity; ICOPE, integrated care for older people; TFA, theoretical framework of acceptability; EHR, electronic health record; NoMAD, normalization measure development questionnaire; SCIROCCO, scaling integrated care in context tool; TIDieR, template for intervention description and replication; ERIC, expert recommendations for implementing change; IPAQ-SF, international physical activity questionnaire-short form.

##### Reach (by residents)

Refers to proportion and representativeness of individuals who are willing to participate in a given initiative (16). The participation rate will be calculated as the proportion of eligible Boon Lay residents who enroll in the program out of all those invited. Representativeness will be assessed by comparing the demographic characteristics of participants (e.g., age, ethnicity, gender) with population data for Boon Lay obtained from the CityPopulation website (https://www.citypopulation.de/).

##### Efficacy (by residents)

Refers to the impact of an intervention on key outcomes (16). The ICOPE program aims to manage declines in IC among community-dwelling older adults (6), and this study will consistently assess its efficacy by measuring the rate of IC change over 12 months from baseline through face-to-face evaluations using ICOPE tools. A recent cohort study reported that the sensitivity of ICOPE screening items for detecting impairments across IC domains ranged from 42.0 to 97.2%, with specificity exceeding 70% for most domains, supporting its reliability (26).

##### Adoption (by providers and residents)

Defined as the intention to try the intervention among early adopters (27). It will be measured through perceived acceptability among providers and residents using an adapted 8-item questionnaire based on the Theoretical Framework of Acceptability (TFA) (28), administered at T0 (baseline) and T2 (6 months). The TFA tool, available in both prospective and retrospective formats for providers and recipients, assesses acceptability across seven constructs: affective attitude, burden, ethicality, intervention coherence, opportunity cost, perceived effectiveness, and overall acceptability. Previous studies have reported a Cronbach’s *α* of 0.86, indicating good internal consistency (29). The resident version will be piloted with a resident advisory group and offered in English and Mandarin, translated using the Brislin’s translation model to ensure accuracy and cultural appropriateness (30). This is particularly important for items such as “There are/were moral or ethical consequences to delivering the ICOPE guide,” as moral reasoning and judgment may vary across diverse ethnic and religious perspectives (31).

##### Implementation (by providers and residents)

Refers to use of intervention as intended (fidelity) and implementation strategies for its adaptation (16). Fidelity of delivery will be assessed among providers via adherence to the intervention protocol, measured using structured observations and the Template for Intervention Description and Replication (TIDieR) checklist from T1 to T3 (32). Fidelity of receipt will be evaluated by tracking adherence to self-monitoring, dietary, and exercise guidelines using EHR data from the ICOPE app and dietary software, supplemented by the 9-item International Physical Activity Questionnaire-Short Form (IPAQ-SF), which is validated in English and Chinese with test–retest reliability of 0.80 and 0.79, respectively (33, 34). Implementation strategies will be monitored using the Expert Recommendations for Implementing Change (ERIC) checklist (35) and 19 ICOPE-specific actions identified from prior Delphi consensus research (36), with validation from a provider focus group at T3, to systematically assess how providers adapt the ICOPE program during implementation.

##### Maintenance (by providers)

Defined as the extent to which a behavior persists six months or more post-intervention (sustainability) and the degree to which a program becomes institutionalized (penetration) (16). Sustainability will be assessed at T3 using the 23-item Normalization Measure Development Questionnaire (NoMAD), which evaluates the extent to which a new practice becomes embedded into routine workflows across four subdomains: coherence, cognitive participation, collective action, and reflexive monitoring, with a total Cronbach’s *α* of 0.89, demonstrating high internal consistency (37). Penetration will be assessed at T3 using the 12-item Scaling Integrated Care in Context (SCIROCCO) tool, which assesses the maturity of integrated care implementation across key domains, supporting knowledge transfer and scalability evaluation, with a total Cronbach’s α of 0.92 (38).

#### Barriers and facilitators of implementation

At T0 (baseline) and T2 (6 months), semi-structured interviews guided by the DoI theory will explore barriers and facilitators to adopting the ICOPE guide among providers and residents (17). For providers, interviews will assess their understanding of implementation purpose for residents (knowledge), the aspects they find appealing or unappealing (persuasion), factors that would motivate them to begin using ICOPE such as screening or comprehensive assessments (decision), the perceived ease or difficulty of integrating ICOPE into practice (implementation), and the influence of colleagues or residents on their adoption decision (social system). For residents, questions will focus on their knowledge of how ICOPE supports their health, what makes it appealing or unappealing, what would encourage participation, the anticipated ease or difficulty of starting ICOPE, and how family, friends, or care staff might affect their decision to engage with components like exercise or health check-ups (Supplementary file 2).

At T3 (12 months), focus groups informed by NPT will examine factors supporting or hindering the sustained use of ICOPE (18). Among providers, discussions will explore how ICOPE aligns with their roles and responsibilities (coherence), what sustains their long-term commitment (cognitive participation), how it has integrated into their daily workflow (collective action), and what has worked well or poorly after one year (reflexive monitoring). For residents, the focus will be on what ICOPE means to them after experiencing it (coherence), what keeps them motivated to continue (cognitive participation), how they have incorporated it into their daily lives (collective action), and the benefits or challenges encountered after one year (reflexive monitoring). Full focus group guides are provided in Supplementary file 3. All these instruments and guides will be piloted in 2–3 providers and residents to help steer the study’s data collection, recruitment and then possibly interpretation of findings.

### Data analysis

#### Quantitative data analysis

Descriptive statistics (means, standard deviations, frequencies) will be used to summarize reach, adoption, implementation fidelity, adaptation strategies, sustainability, and penetration across their respective timepoints. Longitudinal trends, including efficacy (T0, T3), adoption (T0, T2), and implementation fidelity (T1–T3), will be analyzed using paired samples t-tests, ANOVA, or appropriate non-parametric alternatives (e.g., the Friedman test) to assess changes over time. These analyses will be carried out using IBM SPSS 31.

#### Qualitative data analysis

Qualitative data will be systematically examined to uncover adoption and normalization processes. Thematic analysis will be conducted by two independent researchers, disagreement will be resolved after discussion. At T0 and T2, data will be analyzed using DoI constructs, such as knowledge, persuasion, and innovation attributes (e.g., complexity, social influence), to identify barriers (e.g., “High complexity deters residents”) and facilitators (e.g., “SGA outreach boosts uptake”). At T3, NPT constructs, such as coherence and collective action, will guide coding to reveal factors sustaining engagement (e.g., “Simplified app use sustains resident engagement”) and areas for improvement (e.g., “Providers need workflow integration”). A longitudinal thematic matrix will organize DoI-derived themes across T0 and T2, tracing evolving adoption patterns (e.g., “Persuasion barriers evolve into implementation challenges”) (39). Additionally, a multiple-case study approach will compare subgroups, including RHS nurses, AAC staff, SGAs, and residents (stratified by age, age, and ethnicity), using DoI data (T0, T2) to explore adoption differences (40). These analyses will be carried out using NVivo 15.

#### Mixed-methods data integration

Quantitative and qualitative findings will be integrated to provide a comprehensive understanding of adoption and normalization. Joint display analysis will combine quantitative trends (e.g., adoption rates from EHR logs) with qualitative insights (e.g., DoI and NPT themes) in structured tables, facilitating direct comparison (41). Convergent analysis will then evaluate the consistency or divergence between data types, identifying areas of alignment or tension (42). An interpretative framework, drawing on RE-AIM and the CFIR, will contextualize DoI findings (focused on adoption) and NPT findings (focused on normalization), grounding results in established implementation science theories (16, 19). The ERIC compilation will be used to explain how strategies connect prior barriers to further outcomes across time points. For example, where complex procedures identified at T1 through CFIR and DoI as a barrier initially hinder fidelity of screening among providers, ERIC indicates that a strategy like simplified workflows could address this complexity, subsequently enhancing fidelity at T2 by improving consistency in implementation (35).

## Discussion

To our knowledge, this study represents the first multistage evaluation of the ICOPE implementation in a multi-ethnic community setting. While prior research has demonstrated the effectiveness of ICOPE screening for detecting declines in IC when conducted by clinical practitioners (8, 43), the acceptability and functionality of the full care pathway—from Step 1 (screening) to Step 4 (individual care plan implementation and monitoring)—in real-world community health systems remain untested. Communities play a pivotal role in sustaining older adults’ intrinsic capacities (44), making it essential to investigate long-term implementation outcomes such as sustainability and penetration in these settings. Guided by the RE-AIM framework, this protocol enables a comprehensive evaluation of ICOPE’s implementation outcomes in community settings by integrating data from questionnaire surveys, structured observations, and EHRs to systematically assess key outcomes, including reach, efficacy, adoption, implementation (including fidelity in delivery and receipt), sustainability, and penetration (16).

This longitudinal mixed-methods protocol examines factors influencing ICOPE implementation across its stages by synthesizing perspectives from multiple stakeholders. Proposed by WHO in 2007, ICOPE remains a relatively novel framework for both providers and residents (6). Guided by the DoI framework and NPT, this study seeks to identify key factors influencing ICOPE implementation during both early adoption and long-term sustainability stages (45). By mapping stakeholder opinions to contextual factors using the CFIR, this approach offers a comprehensive understanding of how individuals interact with their environments during the intervention. This is a significant consideration, as the empowerment of older adults and their connection to available community resources are crucial for enhancing their intrinsic capacities (46). While prior studies often adopt a single perspective and lack systematic assessment (47), this protocol emphasizes the involvement of multiple stakeholders, integrates their insights, and builds on evidence of ICOPE’s predictive power and implementation challenges.

Identifying effective implementation strategies for ICOPE is essential, as successful implementation requires tailored approaches that account for the unique characteristics of different settings (48). Although a Delphi consensus study has proposed essential ICOPE implementation strategies (36), their effectiveness remains debated. For instance, while Sum et al. (8) highlight barriers for those with low literacy or limited ability to use mobile devices, Briggs et al. (36) argue that digital systems enhance self-management among older adults. By adding descriptive evaluative data on these strategies from a real-world setting, this study can contribute to understanding how ICOPE may be optimally implemented within communities and help develop an evidence-based implementation blueprint. This contribution could guide future adaptations of ICOPE, ensuring its practical utility across diverse community contexts. Beyond ICOPE, we anticipate that the findings of this study will be informative in the context of other interventions and programs designed to be implemented at community level; and a range of traditional population health programs addressed at older adults that are shifting toward community implementation through outreach implementation approaches – e.g., screening for long term conditions, such as cardiovascular disease, or cancers, vaccination programs and similar. Comparative analysis of the implementation findings of this study with existing and emerging implementation studies for other such programs [e.g. Martinengo et al. (49)] will broaden the potential usefulness of this study and make ICOPE implementation a useful ‘implementation case study’ for future reference.

## Conclusion

The novel insights from this multistage evaluation of the ICOPE program will inform international, national, and regional strategies and policies to address the current challenges of sustaining IC among older adults in community settings. Additionally, lessons learned from ICOPE implementation will be integrated into existing healthcare and community services in Singapore, enhancing the quality and effectiveness of interventions available to older residents and their providers. Furthermore, participants and their families will benefit significantly through improved access to tailored care pathways and support for self-management, offering a model that can be adapted by any community aiming to promote healthy aging.

### Strength and limitations

This protocol’s strengths stem from its innovative multistage design and the integration of multiple theoretical frameworks, including RE-AIM, DoI, NPT, and CFIR, which together provide a robust and systematic approach to evaluating ICOPE implementation. The longitudinal mixed-methods design captures quantitative outcomes such as adoption rates alongside qualitative insights like stakeholder experiences, ensuring a comprehensive understanding of barriers and facilitators across diverse community settings. Engaging multiple stakeholders, such as healthcare professionals, non-professional providers, and older residents, fosters a holistic perspective that enhances the relevance of findings for policy and practice. Potential limitations include selection bias, such as over-representation of motivated providers or robust older adults, which may overestimate acceptability. These will be addressed using purposive sampling across provider roles and sensitivity analyses to assess robustness. The small provider sample may also restrict generalizability and statistical power, though this is mitigated by the depth of qualitative data and purposive sampling for diversity. Subsequent larger scale implementation studies, including implementation trials, will be required to provide definitive evaluations of implementation effectiveness of the strategies used to implement ICOPE across Singaporean communities.
