# Supplementary material for: Implementing the ICOPE program amongst community-dwelling older adults in Singapore: a multistage implementation study protocol
Source: Front Public Health. 2025 Dec 12;13:1672852. doi: 10.3389/fpubh.2025.1672852 (PMC12742323; doi:10.3389/fpubh.2025.1672852)
Supplement: Supplementary file 1 [file Table_1.docx]

# Table 1 TFA Generic Questionnaire (Adoption assessment, for both providers and residents)

| TFA construct | Statement for providers | Statement for residents |
| --- | --- | --- |
| **Affective attitude**  *How an individual feels about the intervention* | How comfortable **do/did** you feel **implementing** the ICOPE program (e.g., screening, individual care planning)?   \| Very uncomfortable \| Uncomfortable \| No opinion \| Comfortable \| Very comfortable \| \| --- \| --- \| --- \| --- \| --- \| \| 1 \| **2** \| **3** \| **4** \| **5** \| | How comfortable **do/did** you feel **participating in** the ICOPE program (e.g., screening, exercising)?   \| Very uncomfortable \| Uncomfortable \| No opinion \| Comfortable \| Very comfortable \| \| --- \| --- \| --- \| --- \| --- \| \| 1 \| **2** \| **3** \| **4** \| **5** \| |
| **Burden**  *The amount of effort required to participate in the intervention* | How much effort **do/did** it take to **implement** the ICOPE program?   \| No effort at all \| A little effort \| No opinion \| A lot of effort \| Huge effort \| \| --- \| --- \| --- \| --- \| --- \| \| 1 \| **2** \| **3** \| **4** \| **5** \| | How much effort **do/did** it take to **participate in** the ICOPE program?   \| No effort at all \| A little effort \| No opinion \| A lot of effort \| Huge effort \| \| --- \| --- \| --- \| --- \| --- \| \| 1 \| **2** \| **3** \| **4** \| **5** \| |
| **Ethicality**  *The extent to which the intervention has good fit with an individual’s value system* | There **are/were** moral or ethical consequences to **implement** the ICOPE program.   \| Strongly disagree \| Disagree \| No opinion \| Agree \| Strongly agree \| \| --- \| --- \| --- \| --- \| --- \| \| 1 \| **2** \| **3** \| **4** \| **5** \| | There **are/were** moral or ethical consequences to **participate in** the ICOPE program.   \| Strongly disagree \| Disagree \| No opinion \| Agree \| Strongly agree \| \| --- \| --- \| --- \| --- \| --- \| \| 1 \| **2** \| **3** \| **4** \| **5** \| |
| **Perceived effectiveness**  *The extent to which the intervention is perceived to have achieved its objective* | The ICOPE program **is likely** **to improve**/**has** improved **your clients’ health**   \| Strongly disagree \| Disagree \| No opinion \| Agree \| Strongly agree \| \| --- \| --- \| --- \| --- \| --- \| \| 1 \| **2** \| **3** \| **4** \| **5** \| | The ICOPE program **is likely** **to** **improve**/**has** improved **your health.**   \| Strongly disagree \| Disagree \| No opinion \| Agree \| Strongly agree \| \| --- \| --- \| --- \| --- \| --- \| \| 1 \| **2** \| **3** \| **4** \| **5** \| |
| **Intervention coherence**  *The extent to which the participant understands how the intervention works* | It **is/was** clear to me how the ICOPE program will help improve **your** **clients’** health   \| Strongly disagree \| Disagree \| No opinion \| Agree \| Strongly agree \| \| --- \| --- \| --- \| --- \| --- \| \| 1 \| **2** \| **3** \| **4** \| **5** \|   **Please tell us more about your views* | It **is/was** clear to me how the ICOPE program will help improve **your** health   \| Strongly disagree \| Disagree \| No opinion \| Agree \| Strongly agree \| \| --- \| --- \| --- \| --- \| --- \| \| 1 \| **2** \| **3** \| **4** \| **5** \|   **Please tell us more about your views* |
| **Self-efficacy**  *A participant’s confidence that they can perform behaviour(s) required to participate in the intervention* | How confident **do/did** you feel about **implementing** the ICOPE program?   \| Very unconfident \| Unconfident \| No opinion \| Confident \| Very confident \| \| --- \| --- \| --- \| --- \| --- \| \| 1 \| **2** \| **3** \| **4** \| **5** \| | How confident **do/did** you feel about **participating in** the ICOPE program?   \| Very unconfident \| Unconfident \| No opinion \| Confident \| Very confident \| \| --- \| --- \| --- \| --- \| --- \| \| 1 \| **2** \| **3** \| **4** \| **5** \| |
| **Opportunity costs**  *The benefits, profits or values that would have to be given up to engage with the intervention* | **Implementing** the ICOPE program **will interfere/interfered** with your other priorities   \| Strongly disagree \| Disagree \| No opinion \| Agree \| Strongly agree \| \| --- \| --- \| --- \| --- \| --- \| \| 1 \| **2** \| **3** \| **4** \| **5** \| | **Participating in** the ICOPE program **will interfere/interfered** with your other priorities   \| Strongly disagree \| Disagree \| No opinion \| Agree \| Strongly agree \| \| --- \| --- \| --- \| --- \| --- \| \| 1 \| **2** \| **3** \| **4** \| **5** \| |
| **General acceptability** | How acceptable **is/was implementing** the ICOPE program to you?   \| Completely unacceptable \| Unacceptable \| No opinion \| Acceptable \| Completely acceptable \| \| --- \| --- \| --- \| --- \| --- \| \| 1 \| **2** \| **3** \| **4** \| **5** \| | How acceptable **is/was participating in** the ICOPE program to you?   \| Completely unacceptable \| Unacceptable \| No opinion \| Acceptable \| Completely acceptable \| \| --- \| --- \| --- \| --- \| --- \| \| 1 \| **2** \| **3** \| **4** \| **5** \| |
| **Note:** Items assessing burden and opportunity costs will be reverse-scored, with a higher score indicating higher acceptability. A correlation matrix will be used to compare the relationships between the scores for each TFA item and the general acceptability item.Pre- and post-intervention acceptability will be analyzed using a paired sample t-test. | | |

# Table 2 NoMAD Tool (Sustainability assessment, only for providers)

| **Part A: About Yourself** | | |
| --- | --- | --- |
| **Question** | **Options** | |
| **1. How many years have you worked for this [name of organization/department]?** | 🞎1-2 years 🞎 3-5 years  🞎6-10 years 🞎11-15 years 🞎More than 15 years | |
| **2. How would you describe your professional job category?** | 🞎 Health 🞎Social care 🞎 Combined Health & Social Care 🞎 Voluntary 🞎 Others | |
| **Part B: General Questions About the Intervention** | | |
| **Question** | **Scale** | |
| **1. When you implement the ICOPE program, how familiar does it feel?** | 0 (Still feels very new) to 10 (Feels completely familiar) | |
| **2. Do you feel the ICOPE program is currently a normal part of your work?** | 0 (Not at all) to 10 (Completely) | |
| **3. Do you feel the ICOPE program will become a normal part of your work?** | 0 (Not at all) to 10 (Completely) | |
| **Part C: Detailed Questions About the Intervention**  For each statement please select an answer that best suits your experience using Option A. If the statement is not relevant to you please select an answer from Option B. | | |
| **Section C1 (*Coherence*)** | | |
| **Statement** | **Option A** | **Option B** |
| **1. I can see how the ICOPE program differs from usual ways of working** | Strongly Agree / Agree / Neither agree nor disagree / Disagree / Strongly Disagree | Not relevant to my role / Not relevant at this stage / Not relevant to the intervention |
| **2. Staff in this organization have a shared understanding of the purpose of the ICOPE program** | Strongly Agree / Agree / Neither agree nor disagree / Disagree / Strongly Disagree | Not relevant to my role / Not relevant at this stage / Not relevant to the intervention |
| **3. I understand how the ICOPE program affects the nature of your own work** | Strongly Agree / Agree / Neither agree nor disagree / Disagree / Strongly Disagree | Not relevant to my role / Not relevant at this stage / Not relevant to the intervention |
| **4. I can see the potential value of the ICOPE program for your work** | Strongly Agree / Agree / Neither agree nor disagree / Disagree / Strongly Disagree | Not relevant to my role / Not relevant at this stage / Not relevant to the intervention |
| **Section C2 (*Cognitive participation*)** | | |
| **Statement** | **Option A** | **Option B** |
| **1. There are key people who drive the ICOPE program forward and get others involved** | Strongly Agree / Agree / Neither agree nor disagree / Disagree / Strongly Disagree | Not relevant to my role / Not relevant at this stage / Not relevant to the intervention |
| **2. I believe that participating in the ICOPE program is a legitimate part of my role** | Strongly Agree / Agree / Neither agree nor disagree / Disagree / Strongly Disagree | Not relevant to my role / Not relevant at this stage / Not relevant to the intervention |
| **3. I am open to working with colleagues in new ways to implement the ICOPE program** | Strongly Agree / Agree / Neither agree nor disagree / Disagree / Strongly Disagree | Not relevant to my role / Not relevant at this stage / Not relevant to the intervention |
| **4. I will continue to support the ICOPE program** | Strongly Agree / Agree / Neither agree nor disagree / Disagree / Strongly Disagree | Not relevant to my role / Not relevant at this stage / Not relevant to the intervention |
| **Section C3 (*Collective action)*** | | |
| **Statement** | **Option A** | **Option B** |
| **1. I can easily integrate the ICOPE program into your existing work** | Strongly Agree / Agree / Neither agree nor disagree / Disagree / Strongly Disagree | Not relevant to my role / Not relevant at this stage / Not relevant to the intervention |
| **2. The ICOPE program disrupts working relationships** | Strongly Agree / Agree / Neither agree nor disagree / Disagree / Strongly Disagree | Not relevant to my role / Not relevant at this stage / Not relevant to the intervention |
| **3. I have confidence in other people's ability to implement the ICOPE program** | Strongly Agree / Agree / Neither agree nor disagree / Disagree / Strongly Disagree | Not relevant to my role / Not relevant at this stage / Not relevant to the intervention |
| **4. Work is assigned to those with skills appropriate to the ICOPE program** | Strongly Agree / Agree / Neither agree nor disagree / Disagree / Strongly Disagree | Not relevant to my role / Not relevant at this stage / Not relevant to the intervention |
| **5. Sufficient training is provided to enable staff to implement the ICOPE program** | Strongly Agree / Agree / Neither agree nor disagree / Disagree / Strongly Disagree | Not relevant to my role / Not relevant at this stage / Not relevant to the intervention |
| **6. Sufficient resources are available to support the ICOPE program** | Strongly Agree / Agree / Neither agree nor disagree / Disagree / Strongly Disagree | Not relevant to my role / Not relevant at this stage / Not relevant to the intervention |
| **7. Management adequately supports the ICOPE program** | Strongly Agree / Agree / Neither agree nor disagree / Disagree / Strongly Disagree | Not relevant to my role / Not relevant at this stage / Not relevant to the intervention |
| **Section C4 (*Reflexive monitoring*)** | | |
| **Statement** | **Option A** | **Option B** |
| **1. I am aware of reports about the effects of the ICOPE program** | Strongly Agree / Agree / Neither agree nor disagree / Disagree / Strongly Disagree | Not relevant to my role / Not relevant at this stage / Not relevant to the intervention |
| **2. The staff agree that the ICOPE program is worthwhile** | Strongly Agree / Agree / Neither agree nor disagree / Disagree / Strongly Disagree | Not relevant to my role / Not relevant at this stage / Not relevant to the intervention |
| 1. **I value the effects that the ICOPE program has had on your work** | Strongly Agree / Agree / Neither agree nor disagree / Disagree / Strongly Disagree | Not relevant to my role / Not relevant at this stage / Not relevant to the intervention |
| **4. Feedback about the ICOPE program can be used to improve it in the future** | Strongly Agree / Agree / Neither agree nor disagree / Disagree / Strongly Disagree | Not relevant to my role / Not relevant at this stage / Not relevant to the intervention |
| **5. I can modify how you work with the ICOPE program** | Strongly Agree / Agree / Neither agree nor disagree / Disagree / Strongly Disagree | Not relevant to my role / Not relevant at this stage / Not relevant to the intervention |

# Table 3 SCIROCCO tool (Penetration assessment, only for providers)

| 1.**Readiness to Change**  0 – No acknowledgement of compelling need to change  1 – Compelling need is recognized, but no clear version or strategic plan  2 – Dialogue and consensus-building underway; plan being developed  3 – Vision or plan embedded in policy; leaders and champions emerging  4 – Leadership, vision and plan clear to the general public; pressure for change  5 – Political consensus; public support; visible stakeholder engagement. |
| --- |
| **2. Structure & Governance**  0 – Fragmented structure and governance in place  1 – Recognition of the need for structural and governance change  2 – Formation of task forces, alliances and other informal ways of collaborating  3 – Governance established at a regional or national level  4 – Roadmap for a change programme defined and broadly accepted  5 – Full, integrated programme established, with funding and a clear mandate. |
| **3. Information & eHealth Services**  0 – Information systems are not designed to support integrated care  1 – Information and eHealth services to support integrated care are being piloted  2 – Information and eHealth services to support integrated care are deployed but there is not yet region wide coverage  3 – Information and eHealth services to support integrated care are available via a region-wide service but use of these services is not mandated  4 – Mandated or funded use of regional/national eHealth infrastructure across the healthcare system  5 – Universal, at-scale regional/national eHealth services used by all integrated care stakeholders. |
| **4. Standardization & Simplification**  0 – No standards in place or planned that support integrated care services  1 – Discussion of the necessity of Information and Communication Technology (ICT) to support integrated care and of any standards associated with that ICT  2 – An ICT infrastructure to support integrated care has been agreed together with a recommended set of information standards – there may still be local variations  3 – A recommended set of agreed information standards at regional/national level; some shared procurements of new systems at regional/national level; some large-scale consolidations of ICT underway  4 – A unified set of agreed standards to be used for system implementations specified in procurement documents; many shared procurements of new systems; consolidated data centres and shared services widely deployed  5 – A unified and mandated set of agreed standards to be used for system implementations fully incorporated into procurement processes; clear strategy for regional/national procurement of new systems; consolidated datacentres and shared services (including the cloud) is normal practice. |
| **5. Funding**  0 – No additional funding is available to support the move towards integrated care  1 – Funding is available but mainly for the pilot projects and testing  2 – Consolidated innovation funding available through competitions/grants for individual care providers and small-scale implementation  3 – Regional/national (or European) funding or PPP for scaling-up is available  4 – Regional/national funding for on-going operations is available  5 – Secure multi-year budget, accessible to all stakeholders, to enable further service development |
| **6. Removal of Inhibitors**  0 – No awareness of the effects of inhibitors on integrated care  1 – Awareness of inhibitors but no systematic approach to their management is in place  2 – Strategy for removing inhibitors agreed at a high level  3 – Implementation Plan and process for removing inhibitors have started being implemented locally  4 – Solutions for removal of inhibitors developed and commonly used  5 – High completion rate of projects & programmes; inhibitors no longer an issue for service development |
| 1. **Population Approach**   0 – Population health approach is not applied to the provision of integrated care services  1 – A population risk approach is applied to integrated care services but not yet systematically or to the full population  2 – Risk stratification is used systematically for certain parts of the population (e.g. high-use categories)  3 – Group risk stratification for those who are at risk of becoming frequent service users  4 –Population-wide risk stratification started but not fully acted on  5 – Whole population stratification deployed and fully implemented |
| **8. Citizen Empowerment**  0 – Citizen empowerment is not considered as part of integrated care provision  1 – Citizen empowerment is recognized as important part of integrated care provision but effective policies to support citizen empowerment are still in development  2 - Citizen empowerment is recognized as important part of integrated care provision, effective policies to support citizen empowerment are in place but citizens do not have access to health information and health data  3 - Citizens are consulted on integrated care services and have access to health information and health data  4 – Incentives and tools exist to motivate and support citizens to co-create healthcare services and use these services to participate in decision-making process about their own health  5 – Citizens are fully engaged in decision-making processes about their health, and are included in decision-making on service delivery and policy-making. |
| **9. Evaluation Methods**    Assessment scale:  0 – No evaluation of integrated care services is in place or in development  1 – Evaluation of integrated care services exists, but not as a part of a systematic approach  2 – Evaluation of integrated care services is planned to take place and be established as part of a systematic approach  3 – Some integrated care initiatives and services are evaluated as part of a systematic approach  4 – Most integrated care initiatives are subject to a systematic approach to evaluation; published results  5 – A systematic approach to evaluation, responsiveness to the evaluation outcomes, and evaluation of the desired impact on service redesign (i.e., a closed loop process). |
| **10. Breadth of Ambition**  Assessment scale:  0 – Integrated services arise but not as a result of planning or the implementation of a strategy  1 – The citizen or their family may need to act as the integrator of service in an unpredictable way  2 – Integration within the same level of care (e.g., primary care)  3 – Integration between care levels (e.g., between primary and secondary care)  4 – Integration includes both social care service and health care service needs  5 – Fully integrated health & social care services. |
| **11. Innovation Management**  0 – No innovation management in place  1 – Innovation is encouraged but there is no overall plan  2 – Innovations are captured and there are some mechanisms in place to encourage knowledge transfer  3 – Formalized innovation management process is planned and partially implemented  4 – Formalized innovation management process is in place and widely implemented  5 – Extensive open innovation combined with supporting procurement & the diffusion of good practice is in place |
| **12. Capacity Building**  0 – Integrated care services are not considered for capacity building  1 – Some systematic approaches to capacity building for integrated care services are in place  2 – Cooperation on capacity building for integrated care is growing across the region  3 – Systematic learning about integrated care and change management is in place but not widely  implemented.  4 – Systematic learning about integrated care and change management is widely implemented;  knowledge is shared, skills retained and there is a lower turnover of experienced staff.  5 – A person-centred learning healthcare system’ involving reflection and continuous improvement. |
