# Supplementary material for: Implementing the ICOPE program amongst community-dwelling older adults in Singapore: a multistage implementation study protocol
Source: Front Public Health. 2025 Dec 12;13:1672852. doi: 10.3389/fpubh.2025.1672852 (PMC12742323; doi:10.3389/fpubh.2025.1672852)
Supplement: Supplementary file 2 [file Table_2.docx]

# Interview Guide

1. **Theoretical basis:**

For providers and community stakeholders, DoI examines how ICOPE screening and interventions spread through adoption stages (knowledge, persuasion, decision, implementation) and innovation attributes (e.g., advantage, complexity), focusing on multidisciplinary teamwork in managing older residents. For residents, DoI assesses initial engagement with ICOPE procedures (e.g., screening, care, follow-up) via adoption stages and social influences (e.g., family, community), emphasizing ethnic-specific beliefs. CFIR supplements DoI by contextualizing teamwork (e.g., Inner Setting, Process) and beliefs (e.g., Outer Setting, Individual Characteristics) across T0 and T2, enhancing insights into barriers and facilitators.

1. **Provider Interview Guide (T0, T2)**

“Hello! Thank you for taking the time to talk with me today. My name is Wang Quan, and I'm part of a team studying the ICOPE program, which helps older adults stay healthy through things like exercise, health checks, and diet. We want to hear your thoughts about it. Since you've had experience working with older adults, your insights are especially valuable in understanding how ICOPE fits into daily care practices. There are no right or wrong answers; we just want to know what you think. This will take about 20-30 minutes, and we'll record it so we don't miss anything, but your name won't be linked to what you say. You can stop anytime if you'd like. Do you have any questions before we begin?”

| **DoI Construct** | **Explanation** | **Provider Questions (T0)** | **Provider Questions (T2)** |
| --- | --- | --- | --- |
| **Knowledge** | Awareness of ICOPE and its purpose. | 1. How did you first find out about ICOPE (e.g., training, colleagues), and what stood out to you?  2. What’s your understanding of ICOPE’s goals for older adults’ health? | 1. How has your understanding of ICOPE evolved after using it for 6 months? What's clearer now?  2. Have there been any 'aha moments' where your understanding of ICOPE shifted or deepened? |
| **Persuasion** | Attitudes toward ICOPE's value and feasibility. | 3. What advantages do you see in ICOPE over your usual methods? (Relative Advantage)  4.How well does ICOPE align with your work routines and team goals? (Compatibility)  5. Does ICOPE seem complicated or straightforward to use based on what you know? (Complexity)  6. Could you try ICOPE on a small scale before fully committing to it? What would that look like? (Trialability)  7. Have others’ experiences with ICOPE influenced your interest? (Observability) | 3. What benefits have you seen from ICOPE that make it better than your previous methods? (Relative Advantage)  4. How has ICOPE fit with your daily work and team objectives? (Compatibility)  5. Has ICOPE been simpler or more complex to use than expected? Why? (Complexity)  6. Did trying ICOPE step-by-step (e.g., screening first) help you adopt it? Why or why not? (Trialability)  7. Have colleagues’ successes with ICOPE affected your approach? (Observability) |
| **Decision** | Choice to adopt ICOPE in practice. | 8. What would convince you to start using ICOPE in your practice (e.g., evidence, team support, demand from residents)?  9. What might make you hesitant or reject ICOPE at this point, and why? | 8. Looking back, what motivated you to adopt or continue ICOPE over 6 months?  9. Have you doubted continuing ICOPE? What triggered that? |
| **Implementation** | Early experiences applying ICOPE. | 10. If you were to adopt ICOPE, what do you think would be the easiest and hardest parts to put into practice?  11. Who or what (e.g., training, technology, team support) do you think you'd rely on to get started with ICOPE? | 10. How has applying ICOPE (e.g., screening, care plans) worked in practice so far?  11. What's helped or hindered you in making ICOPE a regular part of your work? |
| **Confirmation** | Reinforcement of applying ICOPE |  | 12. After 6 months, do you feel confident that ICOPE is worth continuing? Why or why not?  13. Have you recommended ICOPE to others (e.g., colleagues, residents)? What prompted that? |
| **Social System/Influence**  **(CFIR Supplement)** | Role of peers, teams, or residents in adoption. | 12. How do you think your team’s communication or roles might affect trying ICOPE?(Inner Setting)  13. How might residents’ needs or community factors shape your use of ICOPE? (Outer Setting) | 14. How has your team’s collaboration or structure impacted ICOPE use? (Inner Setting)  15. How have residents’ feedback or community factors influenced your ICOPE delivery? (Outer Setting) |
| **Future Recommendations** | Ideas to improve and sustain ICOPE | 14. What one change would you suggest for ICOPE to work better for you? | 16. What improvement would make ICOPE more effective in your work?  17. What advice would you give a colleague new to ICOPE? |

1. **Interview guide for residents (T0, T2)**

“Hello! Thank you for taking the time to speak with me today. My name is Wang Quan, and I'm part of a team studying the ICOPE program, which supports older adults' health through screening and interventions. We're interested in how programs like ICOPE might improve daily well-being for people like you. There are no right or wrong answers; we just want to understand your perspective. This will take about 20-30 minutes, and we'll record it for accuracy, but your name won't be linked to your responses. You can stop anytime if you'd like. Do you have any questions before we begin?”

| **DoI Construct** | **Explanation** | **Interview Questions by T0** | **Interview Questions by T2** |
| --- | --- | --- | --- |
| **Knowledge** | Awareness of ICOPE and its benefits. | 1. What have you heard about the ICOPE program and how it might support your health as you age?  2. How did you learn about ICOPE (e.g., staff, family, flyers), and what caught your attention? | 1. After 6 months, what do you now know about ICOPE and how it helps you (e.g., with strength, memory)?  2. What have you learned from doing ICOPE or hearing about it from others? |
| **Persuasion** | Attitudes toward trying ICOPE. | 3. What do you think ICOPE could do for you that's better than what you're doing now to stay healthy? (Relative Advantage)  4. Does ICOPE seem like something that fits with your daily life and preferences? (Compatibility)  5. Does ICOPE sound simple or confusing to you based on what you've heard? (Complexity)  6. Would you be willing to try ICOPE a little at a time (e.g., one screening) to see how it goes? (Trialability)  7. Have you seen or heard about other people like you using ICOPE, and does that make you curious? (Observability) | 3. What's been the best part of ICOPE for you compared to how you managed your health before? (Relative Advantage)  4. Has ICOPE felt like a good fit for your daily routine and needs these past 6 months? (Compatibility)  5. Has ICOPE been easier or trickier to do than you thought? What's made it that way? (Complexity)  6. Did starting ICOPE slowly (e.g., one step at a time) make it easier to keep going? (Trialability)  7. Have you seen others benefit from ICOPE, and has that changed how you feel about it? (Observability) |
| **Decision** | Choice to participate in ICOPE. | 8. What would encourage you to join ICOPE and start doing things like check-ups or exercises?  9. What might hold you back from trying ICOPE, and why? | 8. What kept you going with ICOPE over these 6 months (e.g., feeling better, encouragement)?  9. Have you ever felt like quitting ICOPE? What made you think that? |
| **Implementation** | Early engagement with ICOPE activities. | 10. If you started ICOPE, what do you think would be easy or hard about doing it (e.g., using an app, going to a center)?  11. What kind of help do you think you'd need to get going with ICOPE (e.g., family, staff, transport)? | 10. How has it been for you to do ICOPE activities (e.g., exercises, check-ups, app use) so far?  11. What's made it easier or harder to stick with ICOPE in your daily life? |
| **Confirmation** |  | NA | 12. After 6 months, do you think ICOPE is worth continuing for your health? Why or why not?  13. Have you told others (e.g., friends, family) about ICOPE? What did you say? |
| **Social System/Influence** | Influence of family, friends, or staff. | 12. How might your family or community’s views affect your interest in ICOPE? (Outer Setting)  13. How do your personal beliefs about health influence your thoughts on ICOPE? (Individual Characteristics) | 14. How have family or community reactions shaped your ICOPE experience? (Outer Setting)  15. How have your health beliefs affected your participation in ICOPE? (Individual Characteristics) |
| **Future Recommendations** | Ideas to improve and sustain ICOPE | 14. What one change would make ICOPE better for you? | 16. What improvement would make ICOPE work better for you?  17. What advice would you give someone new to ICOPE? |
