# Supplementary material for: Implementing the ICOPE program amongst community-dwelling older adults in Singapore: a multistage implementation study protocol
Source: Front Public Health. 2025 Dec 12;13:1672852. doi: 10.3389/fpubh.2025.1672852 (PMC12742323; doi:10.3389/fpubh.2025.1672852)
Supplement: Supplementary file 3 [file Table_3.docx]

# Focus Group Guide

**1.Theoretical basis:**

For providers and community stakeholders, NPT assesses how ICOPE screening and interventions are normalized in practice through coherence (understanding its purpose), cognitive participation (sustaining engagement), collective action (integrating into workflows), and reflexive monitoring (evaluating outcomes), with a focus on multidisciplinary teamwork. For residents, NPT explores how ICOPE embeds in daily life via coherence (grasping its role), cognitive participation (maintaining commitment), collective action (adopting routines), and reflexive monitoring (assessing benefits), emphasizing ethnic-specific beliefs. CFIR supplements NPT by providing contextual depth across T3, enhancing teamwork insights (e.g., Inner Setting, Process) and ethnic belief perspectives (e.g., Outer Setting, Individual Characteristics).

**2. Focus group guide for providers (T3)**

“Thank you for joining us today! You've been involved with the ICOPE program for a year now. We'd like to hear your ideas on how to keep it going and improve it as providers. We'll discuss how it fits into your work and what would help you sustain it. Feel free to share anything that comes to mind!”

| **NPT Construct** | **Explanation** | **Focus Group Questions (T3)** |
| --- | --- | --- |
| **Coherence** | Providers' work to understand ICOPE's role in promoting or inhibiting routine care delivery. | 1. After a year, how does ICOPE’s purpose fit into your long-term care goals for older adults? 2. Which ICOPE elements (e.g., screening, care plans) feel most integrated into your practice now? 3. How has ICOPE’s role in your work settled over time, and what shaped that? |
| **Cognitive Participation** | Providers' work to enroll themselves and others in delivering ICOPE sustainably. | 1. How do you get yourself or your team engaged in using ICOPE consistently, despite any hurdles? 2. What would make you more willing—or reluctant—to keep delivering ICOPE moving forward? |
| **Collective Action** | Providers' work to enact ICOPE in daily practice and teamwork. |  |
| -Contextual Integration (CI) | Work to align ICOPE with organizational resources and systems. | 1. How does ICOPE fit with your workplace resources (e.g., staff availability, leader support) after a year? 2. What system-level factors (e.g., policies, funding) support or block ICOPE becoming routine? |
| - Relational Integration (RI) | Work to build trust and collaboration for ICOPE delivery. | 1. How has ICOPE shaped your trust and teamwork with others (e.g., NUP staff, AAC managers)? 2. What fosters or disrupts your collaboration with colleagues or partners in delivering ICOPE? |
| - Interactional Workability (IW) | Work to make ICOPE practical in resident interactions. | 1. How manageable is it to use ICOPE guide (e.g., screening, following-up) in your day-to-day resident interactions? 2. What adjustments have you made to streamline ICOPE tasks in practice? |
| - Skill Set Workability (SSW) | Work to match skills to ICOPE tasks. | 1. How well do your skills match ICOPE tasks (e.g., assessments, planning)? 2. What additional training or support help you enact ICOPE more confidently? |
| **Reflexive Monitoring** | Providers' work to appraise ICOPE's effects and refine its use. | 1. What successes or difficulties have you noticed with ICOPE's impact on residents' health or your workflow after a year? 2. How do your team evaluate ICOPE's value, and how does that affect your thoughts? |
| **CFIR Supplement** | Context for teamwork and sustainability | 1. How do your team’s dynamics or communication affect keeping ICOPE going? (Inner Setting) 2. How do community or resident factors influence ICOPE’s long-term use? (Outer Setting) |
| **Future Recommendations** | Ideas to improve/sustain ICOPE | 1. What change would help ICOPE remain sustainable in your practice long-term? 2. What advice would you give to sustain ICOPE for other providers? |

**2. Focus group guide for residents (T3)**

“Thank you for joining us today! You've been part of the ICOPE program for a year now. We'd like to hear your ideas on how to make it better and keep it going for people like you. We'll talk about how it fits into your life and what would help you stick with it. Feel free to share anything that comes to mind!”

| **NPT Construct** | **Explanation** | **Focus Group Questions (T3)** |
| --- | --- | --- |
| **Coherence** | Residents' work to understand ICOPE's role in promoting or inhibiting routine self-care. | 1. After a year, how does ICOPE’s role feel as part of your usual self-care? 2. Which ICOPE activities (e.g., exercises, check-ups) now feel most natural in your routine? 3. How has ICOPE’s place in your life settled over time, and what influenced that? |
| **Cognitive Participation** | Residents' work to enroll themselves and others in following ICOPE sustainably. | 1. How do you or others (e.g., family, friends) keep you involved in sticking with ICOPE over time? 2. What would make you more eager—or less likely—to keep following ICOPE in the future? |
| **Collective Action** | Residents' work to enact ICOPE in daily routines and interactions. |  |
| -Contextual Integration (CI) | Work to fit ICOPE into personal resources and context. | 1. How does ICOPE blend with your home life or resources (e.g., time, space, access to AAC programs)? 2. What in your daily context (e.g., transport, family) makes ICOPE easier or tougher to follow? |
| - Relational Integration (RI) | Work to use relationships to support ICOPE participation. | 1. How do family, friends, or staff support your efforts with ICOPE? 2. What relationships make ICOPE feel more trustworthy or challenging for you? |
| - Interactional Workability (IW) | Work to make ICOPE tasks practical in daily life. | 1. How easy is it to do ICOPE tasks (e.g., exercises, check-ups) as part of your daily life? 2. What adjustments have you made to fit ICOPE activities into your routine more smoothly? |
| - Skill Set Workability (SSW) | Work to apply abilities to ICOPE tasks. | 1. How comfortable are you with your ability to handle ICOPE tasks (e.g., using apps, exercising)? 2. What help (e.g., simpler instructions) would make ICOPE tasks feel more doable? |
| **Reflexive Monitoring** | Residents' work to appraise ICOPE's effects and adjust participation. | 1. What successes or difficulties have you noticed with ICOPE's impact on your health or daily life after a year? 2. How do your families or friends evaluate ICOPE's value to you, and how does that affect your thoughts? |
| **CFIR Supplement** | Context for beliefs and sustainability | 1. How do your community or cultural background affect sticking with ICOPE? (Outer Setting) 2. How do your personal health beliefs shape your ICOPE experience? (Individual Characteristics) |
| **Future Recommendations** | Ideas to improve/sustain ICOPE | 1. What change would help you keep ICOPE going in your life long-term? 2. What advice would you give to keep ICOPE going for others like you? |
